# Supplementary material for: Reproducibility in cytometry: Signals analysis and its connection to uncertainty quantification
Source: PLoS One. 2023 Dec 22;18(12):e0295502. doi: 10.1371/journal.pone.0295502 (PMC10745152; doi:10.1371/journal.pone.0295502)
Supplement: S1 Appendix — (PDF) [file pone.0295502.s001.pdf]

A Optimization for Multiplet Deconvolution

In this appendix we describe the mathematical formulation and solution of optimization for multiplet deconvolution. See Ref. [1] for background on the underlying mathematical methods. For convenience, we restate the key equations underlying this problem. Specifically, the objective is given by

$$\mathcal{L}_d = \sum_{j=1}^{\mathcal{M}} \Delta\hat{\mathfrak{f}}_j^{\mathrm{T}} \; \Xi^{-1} \, \Delta\hat{\mathfrak{f}}_j + \Delta\chi_j^{\mathbf{T}} \Upsilon^{-1} \Delta\chi_j, \tag{41}$$

whereas the constraint in the complex basis is

$$\hat{\mathbf{m}} = \sum_j \mathbf{\Lambda}^{-1}(c_j, v_j, R_j, \Delta t_j)[\bar{\mathbf{f}} + \Delta\hat{\mathbf{f}}_j], \tag{42}$$

where $\bar{\mathbf{f}}$ is the complex representation of $\bar{\mathfrak{f}}$. Our goal is to convert Eq. (42) to the same basis as $\hat{\mathfrak{f}}$ and then simplify the optimization problem implied by Eq. (41).

Define $\mathbf{\Lambda}_j^{-1} = \mathbf{\Lambda}^{-1}(c_j, v_j, R_j, \Delta t_j)$. Next, note that a vector $\hat{\mathbf{f}}$ has $2M+1$ elements. Because we compute them via a DFT, we take the convention that the first $M+1$ modes correspond to $k = 0, \pi, 2\pi, ..., M\pi$, whereas the last M modes correspond to $k = -M\pi, -(M-1)\pi, ..., -\pi$. Moreover, because the signals are real in the time-domain, we know that $\hat{f}(k) = \hat{f}(-k)^{\star}$, where $\star$ denotes the complex conjugate. This implies that all of the relevant information about $\hat{\mathbf{f}}$ is contained in $\hat{\mathfrak{f}}$, since only M complex Fourier modes are needed to describe the signal. To see this explicitly, express an arbitrary $\hat{\mathbf{f}}$ as

$$\hat{\mathbf{f}} = \begin{pmatrix} \mathfrak{a}_1 \\ \mathfrak{a}_2 + i\mathfrak{a}_{M+2} \\ \mathfrak{a}_3 + i\mathfrak{a}_{M+3} \\ \vdots \\ \mathfrak{a}_{M+1} + i\mathfrak{a}_{2M+1} \\ \mathfrak{a}_{M+1} - i\mathfrak{a}_{2M+1} \\ \mathfrak{a}_M - i\mathfrak{a}_{2M} \\ \vdots \\ \mathfrak{a}_2 - i\mathfrak{a}_{M+2} \end{pmatrix}, \tag{43}$$

where $\mathfrak{a}_j$ is the jth element of $\hat{\mathfrak{f}}$.

To derive the transformed version of $\mathbf{\Lambda}$ in the basis of $\hat{\mathfrak{f}}$, first decompose the matrix into blocks via

$$\mathbf{\Lambda} = \begin{bmatrix} A & B \\ C & D \end{bmatrix}, \tag{44}$$

where A is $(M+1)\times(M+1)$, B is $(M+1)\times M$, C is $M\times(M+1)$, and D is $M\times M$. Clearly A couples the first $M+1$ modes of $\hat{\mathbf{f}}$ into one another, B couples the remaining M modes into the first $M+1$, and so forth. However, because the signal remains real in the time-domain after transformation by $\mathbf{\Lambda}$, knowledge of A and B is sufficient to determine the transformation matrix in the basis of $\hat{\mathfrak{f}}$. Let $\mathcal{F}$ denote that operator that reverse the order of columns in a matrix, $\mathcal{T}_c$ ($\mathcal{T}_r$) denote the operator that removes the first column (row) of a matrix, and $\mathbf{0}_M$ denote a column vector with M zeros. Then it is straightforward to show that the operator $\mathbf{\Lambda}$ transforms to

$$\mathbf{\Theta} = \begin{bmatrix} \tilde{A} & \tilde{B} \\ \tilde{C} & \tilde{D} \end{bmatrix}, \tag{45}$$

where

$$\begin{aligned} \tilde{A} &= \Re(A) + [\mathbf{0}_{M+1}, \mathcal{F}(\Re(B))], \\ \tilde{B} &= -\mathcal{T}_c(\Im(A)) + \mathcal{F}(\Im(B)), \\ \tilde{C} &= \mathcal{T}_r(\Im(A)) + [\mathbf{0}_M, \mathcal{F}(\Im(B))], \\ \tilde{D} &= \mathcal{T}_r(\Re(A)) - \mathcal{F}(\Re(B)), \end{aligned} \tag{46}$$

and $\Re$ and $\Im$ denote the real and imaginary components. Thus we arrive at an expression for $\hat{\mathbf{m}}$ expressed in the basis of $\hat{\mathfrak{f}}$, viz,

$$\hat{\mathfrak{m}} = \sum_j \mathbf{\Theta}_j^{-1}[\bar{\mathfrak{f}} + \Delta\hat{\mathfrak{f}}_j]. \tag{47}$$

Minimizing Eq. (41) subject to Eq. (47) may entail optimizing over $\mathcal{O}(100)$ or more variables corresponding to: (i) the scale parameters c, v, R, and Δt; and (ii) the real and imaginary parts of the mode-weights. The latter comprise the majority of variables, although they only appear up to second order. In contrast, the transformation variables, while few in number, appear in highly non-linear function associated with the matrix $\mathbf{\Theta}^{-1}$ in Eq. (47). Further compounding these issues is the fact that both $\mathbf{\Theta}^{-1}$ and Ξ are dense matrices, the latter possibly having eigenvalues close to zero. This may yield a relatively large numerical problem that is poorly scaled, and thus challenging to solve.

Fortunately, the constraint given by Eq. (42) is linear in the mode-weights, which yields a key simplification. Without loss of generality, one finds

$$\Delta\hat{\mathfrak{f}}_1 = \mathbf{\Theta}_1 \left[\hat{\mathfrak{m}} - \mathbf{\Theta}_1^{-1}\bar{\mathfrak{f}} - \sum_{j=2}^{\mathcal{M}} \mathbf{\Theta}_j^{-1}[\bar{\mathfrak{f}} + \Delta\hat{\mathfrak{f}}_j] \right]. \tag{48}$$

Equation (48) can be substituted into Eq. (39) and minimization performed over the remaining modes $\Delta\hat{\mathfrak{f}}_j$ for $j \geq 2$ for fixed transformation parameters associated with the $\mathbf{\Theta}_j$. We leave this exercise for the reader. For the case of doublets, one find that

$$\begin{aligned} G &= \mathbf{\Theta}_1 \left[\hat{\mathfrak{m}} - (\mathbf{\Theta}_1^{-1} + \mathbf{\Theta}_2^{-1})\bar{\mathfrak{f}} \right], \\ \Delta\hat{\mathfrak{f}}_2^* &= \left[(\mathbf{\Theta}_1\mathbf{\Theta}_2^{-1})^{\mathrm{T}}\Xi^{-1}\mathbf{\Theta}_1\mathbf{\Theta}_2^{-1} + \Xi^{-1} \right]^{-1} \mathbf{\Theta}_1\mathbf{\Theta}_2^{-2}\Xi^{-1}G, \\ \Delta\hat{\mathfrak{f}}_1^* &= \mathbf{\Theta}_1[G - \mathbf{\Theta}_2^{-1}\Delta\hat{\mathfrak{f}}_2], \end{aligned}$$

where $\Delta\hat{\mathfrak{f}}_2^*$ and $\Delta\hat{\mathfrak{f}}_1^*$ are the optimal mode perturbations (the $*$ is distinct from the complex conjugate $\star$). Having the $\Delta\hat{\mathfrak{f}}_j^*$ in terms of the transformation parameters (via the $\mathbf{\Theta}_j$), we may then express the objective as

$$\mathcal{L}_d = \sum_j \left[\Delta\hat{\mathfrak{f}}_j^* \right]^{\mathrm{T}} \Xi^{-1}\Delta\hat{\mathfrak{f}}_j^* + \Delta\chi_j^{\mathbf{T}}\Upsilon^{-1}\Delta\chi_j. \tag{49}$$

This $\mathcal{L}_d$ can then be optimized as a function of the scale transformations parameters.

Reference

1. Arfken GB, Weber HJ, Harris FE. Mathematical Methods for Physicists: A Comprehensive Guide. Elsevier Science; 2013.
